# Silence to grow: psychological transformation during long-time engagement in green and blue nature

**DOI:** 10.3389/fpsyg.2026.1652406

**Published:** 2026-03-16

**Authors:** Helga Synnevåg Løvoll, Gunvor Marie Dyrdal

**Affiliations:** 1 Department of sports, physical education and outdoor studies, Volda University College, Volda, Norway; 2 Norwegian University of Science and Technology, Psykolog Dyrdal, Oslo, Norway

**Keywords:** aesthetic and creative environment, awareness, awe experiences, bodily rhythm, flexibility hypotheses of healing, growth-oriented mindset, loneliness mindset, oceanic expeditions

## Abstract

This study investigates the influence of extended nature engagement on psychological transformation within a Norwegian undergraduate outdoor life and nature guide program. We examined two distinct immersive experiences: a 1-month tall ship adventure where students volunteered in a social entrepreneurship program for youth, and a solo forest hike of up to 14 days incorporating Shinrin Yoku-inspired invitations. Data included post-experience interviews with four students (two from each group) and analyses of their written exam papers, which partly also documented systematic wellbeing monitoring. As hypothesized, participants in both groups experienced an emotional “dip” or challenging low point, which proved essential for the transformation and emergence of new perspectives. Developing a solitude mindset over a loneliness mindset was a main challenge for the solo hikers. Five overarching themes were identified: 1. Reaching a mental low point before growth, 2. Time to reflect, 3. Being present, 4. The role of nature and awe experiences and 5. Bodily rhythm and awareness. The study suggests that prolonged nature engagement offers significant potential for human growth and development, although specific intervening factors may affect the outcomes. Nevertheless, the inherent capacity for human transformation within such experiences is evident.

## Introduction

Seeking solitude in the wilderness for personal development is far from a new phenomenon. For centuries, people have turned to nature for clarity, meaning, and transformation. In contemporary society, this impulse has gained renewed relevance, particularly through activities such as long-distance hiking, pilgrimage, solo experiences in natural settings, and increasingly, oceanic expeditions. Long-distance journeys in nature offer participants a chance to withdraw from daily demands and engage in both an outer and an inner journey. Moreover, being in a facilitated outdoor learning environment have shown to have meaningful, transformative, personally relevant, and learning promotive characteristics (Taniguchi, 2004).

Long-distance walking, often undertaken in solitude, has emerged as a particularly potent catalyst for personal transformation for tourists as well as nature guides (Saunders et al., 2013). Walking in solitude can promote increased self-awareness, a sense of meaning and the development of a growth-oriented mindset. In their study, Saunders et al. (2013) found that personal development was also the main motivation for becoming long-distance guides. Moreover, long-distance walking offers mental health benefits through physical exertion, solitude, and immersion in nature (Mau et al., 2021). Mau et al. (2023a) argue that such journeys can lead to a process of “becoming a person,” where individuals confront existential questions and develop a deeper sense of self. Inspired by Rollo May, one can view nature as silent and inorganic—a standpoint that foregrounds *nothingness*. In this view, nature has a mirroring effect, inviting a temporary loosening of the sense of self (May, 1953). Such an encounter may provide a starting point for reorienting our understanding of who we are and where we are heading. This does not imply that there is an inherent association between long-distance walking and positive outcomes, but it holds a potential for personal growth and flourishing. While chosen solitude can be beneficial, it can also entail risk, particularly when it shifts toward social isolation (Bingham, 2023). Negative experiences of solo trips could also result from experiencing challenging weather conditions, being unprepared for the actual experience of being alone in nature, and prolonged stays. Hence, a fine-grained understanding of growth-related elements is necessary.

In the blue element, attention to longer sailing expeditions has gained increased attention (Schijf et al., 2017). In their systematic review, Schijf et al. (2017) found that participating youths reported changes in feelings of overall self-worth and in their ability to form social relationships and create friendships. From retrospective research interviews on the significance of tall ship sailing, participants point to the character-building and virtue developing process, sparked by the sailing adventure (Marshall et al., 2020). In other words, there have been transcending growth experiences, pointing to existential dimensions of being human. This resonates with the “flexibility hypotheses of healing,” suggesting that “conveying a sense of change, healing rituals shift sufferers’ mode of being-in-the-world, including their cognitive, emotional, and physical state or stance, toward openness to new ways of being” (Hinton and Kirmayer, 2017, p. 4). The ability to shift attention, as well as producing images, metaphors or other representations emerge in an aesthetic and creative environment. While this flexibility mindset has mainly been studied within the context of musical experiences, it seems relevant for personal transformational processes emerging during long-time nature engagement. Løvoll and Løvoll (2025) argue that a *mindshift* is needed to deal with fundamental root causes of modeling a sustainable future society. This includes new perspectives on what is valuable and meaningful in a perspective that exceeds individual needs. Nature and the arts can serve as important domains for transcending growth, propelled by complex emotions, such as interest, awe, wonder, and love, emotions that are often evoked through well-facilitated engagement with nature and artistic processes (Løvoll and Løvoll, 2025). Such emotions are identified as “opportunity emotions,” emotions that play a significant role in psychological growth (Vittersø, 2025).

Reiss (2021) provides an ethnographic perspective navigating both the internal and societal landscapes during long-distance hiking. Stripped of the “facade” of everyday routines and social roles, hikers are often compelled to confront a more authentic or core sense of self. This condition, combined with the rhythm and simplicity of walking, creates a space for introspection and identity reconstruction. Moving beyond the “facade” seems to be relevant in the tall ship environment as well, where privacy is limited and the only people you meet while sailing are your fellow crew members.

In a study of adolescents at risk of social drop-out and societal exclusion, informants talked about transformative experiences of a sailing expedition, where commitment, social wellbeing, familiarization of seamanship, and self-acceptance were common themes (Dyrdal and Løvoll, 2023). In a follow-up investigation identifying valuable moments of transformative experiences during the tall ship experience, moments of silence and time alone was particularly important (Løvoll and Dyrdal 2025). The context where these experiences emerged, alone on lookout or steering duty, away from social media and daily habits, offered a new way of being. During this social media “detox” experience, participants became aware of personal resources as well as their core values in life. To gain a deeper understanding of the psychological processes activated during prolonged nature exposure, more studies are needed. The lives of individuals living in modern, industrialized, Western societies, are often defined by activities, busyness, and effectiveness. Aspect of silence, presence of nature, breaking daily routines to allow for unplanned time, and the experience of *being* rather than *acting* are important and existential ways of being and essential dimensions of wellbeing. In this study we explore personal transformation and growth within a nature context, defined by both blue and green environments. These contexts invite possible transcending experiences where participants can discover deep and profound insights in own life (Dyrdal and Løvoll, 2023), spiritual awareness (Løvoll and Sæther, 2022), or a transformation of mindset, understood as “creative wellbeing” (Torrissen and Løvoll 2025). Creative wellbeing is an interdisciplinary theory, explaining how creative processes stimulated by nature and the arts spark a willingness to change perspectives and to live in accordance with core values. Through complex emotions one can reestablish connection with existential dimensions of ourselves in the world, which can guide us to new images and insights of how to live our lives. However, the paths to these new perspectives and insights challenge the idea that happiness is easy and comfortable. Growth involves struggle and hardship, and as such, long-time engagement in nature as a transformational experience could include every kind of emotion.

In this article, we explore transcendence through resilience as an important aspect of personal growth during long-time nature engagement. We aim to identify the contextual and situational conditions under which individuals adopt resilience as a deliberate stance when encountering emotional, social, or physical challenges in outdoor settings. Resilience here is defined and understood as “the process and outcome of successfully adapting to difficult or challenging life experiences, especially through mental, emotional, and behavioral flexibility and adjustment to external and internal demands” (APA, 2026).

### The role of wilderness and oceanic landscapes

Nature-based physical activity has been shown to positively impact mental health. In five studies, Wolsko et al. (2019) document that such activities can reduce symptoms of anxiety and depression while increasing life satisfaction and overall wellbeing. The stress reducing effect of dwelling in natural environment, found in Hartig et al. (1991) has been confirmed in many studies. Insights into how humans engage with nature at an existential level are further deepened in the body of literature on solitary nature experiences. Potter and O’Connell (2005) describe how solo experiences are used in educational programs to foster independence, reflection, and emotional development. Petersen et al. (2021) confirm these findings in a cross-cultural education study, observing that being alone in nature can enhance wellbeing, reduce stress, and deepen one’s connection to both self and surroundings. Importantly, the oceanic landscape offers a parallel yet distinct setting for personal transformation. Time alone time, combined with shared responsibilities and task synchronization were typical characteristics in an anthropological investigation of sailing trainees, offering opportunities for transformative experiences (Montserrat, 2020). These seascapes, like terrestrial wilderness, provide a liminal space where individuals can disconnect from societal roles and reconnect with their own inner selves. The COVID-19 pandemic highlighted how dwelling in nature became an important coping strategy. Mau et al. (2023b) explored how solitary walks in nature helped older adults to manage feelings of loneliness during lockdowns. These walks provided not only physical activity but also emotional comfort and a sense of continuity in uncertain times.

The broader relationship between nature and happiness is well-documented. MacKerron and Mourato (2013) demonstrated that people report higher levels of happiness when in natural environments compared to urban settings. Similarly, Hakoköngäs and Puhakka (2023) found that Finnish adolescents associate nature with happiness, peace, and emotional balance, suggesting that this connection is both intuitive and culturally embedded. In sum, nature serves as a multifaceted space for personal growth. Yet, as research shows, growth often requires navigating discomfort and vulnerability. In an era of constant connectivity and information overload, nature remains one of the few settings where silence, struggle, and self-renewal are still possible. Taken together, the research suggests that both wilderness and oceanic landscapes serve as powerful arenas for physical and psychological renewal. Whether through long-distance hiking, or tall ship voyages, nature provides a space to disconnect from external pressures and turn inward.

### Being and doing: exploring existential questions while in nature

The interplay between “being” and “doing” is central to understanding how individuals experience wellbeing and growth, particularly in natural environments. While “doing” often refers to action-oriented engagement—such as hiking or exploring—“being” encompasses a deeper sense of presence, reflection, and connection. Recent research suggests that both dimensions are essential for fostering autonomy, identity, and eudaimonic wellbeing. Sheldon et al. (2021) emphasize the importance of self-supported autonomy, arguing that individuals who actively maintain their own autonomy experience greater motivation and wellbeing than those who rely solely on external validation. This internal orientation aligns with the concept of “being,” where individuals cultivate resilience by self-awareness and intrinsic motivation, often facilitated by time in nature.

Nature itself serves as a powerful context for both being and doing. Pritchard et al. (2020) conducted a meta-analysis showing a strong relationship between nature connectedness and eudaimonic wellbeing, highlighting how meaningful engagement with nature supports personal growth and life purpose. Similarly, Olivos and Clayton (2016) argue that a strong environmental identity and sense of connectedness to nature are key contributors to quality of life, reinforcing the idea that nature is not just a backdrop for activity but a partner in psychological development.

Mountain hiking, as explored by Próchniak (2022), illustrates how physical activity in nature can foster distinct wellbeing profiles. “Hard” hikers, who seek challenge and achievement, and “soft” hikers, who prioritize contemplation and connection, both derive benefits—suggesting that doing and being are not mutually exclusive but rather complementary pathways to wellbeing.

The therapeutic potential of natural landscapes is further supported by Brooke and Williams (2021), who describe Iceland’s wilderness as a “therapeutic landscape.” These white, open spaces offer a sense of freedom and emotional release, allowing individuals to process experiences and reconnect with themselves. Brooks et al. (2017) also found that mood improvements from nature contact vary by season and type, indicating that both the quality and context of nature experiences influence emotional outcomes. In sum, the integration of being and doing in natural settings supports a holistic model of wellbeing (Vittersø, 2025), including the importance of our relational needs, providing care to other humans as well as other living organisms. Whether through active exploration or quiet reflection, nature provides a space where individuals can align with their values, support their autonomy, and cultivate a deeper sense of self.

### Solitude and loneliness: distinct experiences with divergent outcomes

Although often used interchangeably, solitude and loneliness represent fundamentally different psychological experiences. Loneliness is typically characterized by a distressing sense of social disconnection, while solitude can be a chosen and enriching state of being alone. Understanding this distinction is crucial for promoting mental wellbeing, especially in contexts where time alone is inevitable or actively chosen. Loneliness is generally associated with negative emotional outcomes, including anxiety, depression, and reduced life satisfaction (Bingham, 2023). It arises from a perceived gap between desired and actual social interaction. In contrast, solitude—when voluntarily chosen—can foster self-reflection, creativity, and personal growth (Naor and Mayseless, 2020b; Zelenski et al., 2021). The key difference lies in perception and intentionality.

Rodriguez et al. (2020) found that cognitive reappraisal—reframing how one interprets time spent alone—can buffer the emotional toll of isolation. Their later work (Rodriguez et al., 2023) further demonstrated that individuals who view solitude positively experience it as restorative rather than distressing, even if they initially feel lonely. This suggests that mindset plays a pivotal role in transforming loneliness into solitude. Personality traits also influence how solitude is experienced. Weinstein et al. (2023) identified individuals high in autonomy, mindfulness, and emotional stability to be more likely to thrive in solitude. Similarly, Zelenski et al. (2013, 2021) found that introverts often report greater wellbeing during solitary experiences, as they are less reliant on external stimulation for emotional fulfillment.

Solitude in nature, particularly through structured experiences like wilderness solos, has been shown to facilitate deep personal transformation. Naor and Mayseless (2020a) describe how peak experiences in nature can lead to lasting shifts in self-perception and values. Williams (2012) emphasize that such experiences, when framed as intentional and meaningful, can promote psychological integration and resilience. Solo expeditions and outdoor recreation also highlight the therapeutic potential of solitude. Smith and Palmer (2015) found that individuals on solo journeys often report increased clarity, emotional release, and a sense of empowerment. Wolsko and Lindberg (2013) link these outcomes to enhanced mindfulness and nature connectedness, which are associated with psychological wellbeing. However, solitude is not universally beneficial. Without the right mindset or context, it can exacerbate feelings of isolation. Fu (2023) notes that tourists who engage in solitude with a focus on self-improvement report higher eudaimonic and hedonic outcomes, while those who feel forced into solitude may experience diminished wellbeing.

In conclusion, solitude and loneliness are not merely two ends of a spectrum but qualitatively different experiences shaped by perception, personality, and context. While loneliness reflects a painful absence, solitude can represent a powerful presence—of self, of nature, and of meaning. Cultivating the ability to reframe and embrace solitude may be a vital skill for enhancing psychological resilience in an increasingly overstimulated world.

### Forest solo experiences and blue sailing adventures

The present study aims to investigate how undergraduate students experience prolonged nature experiences. They chose either a solo hike in a Norwegian forest national park or sailing in international waters for 1 month as a tall ship sailing crew. The duration of the forest solo trips were up to 14 days, in solitude, in a remote area covered by pine trees, with no support from others. To actively promote attention to nature connection, we used invitations from Shinrin Yoku practice as voluntary exercises and experiments. Shinrin Yoku comes from a Japanese practice of linking mindfulness and nature connection through immersive experiences in nature. A systematic review on Shinrin Yoku found this practice effective in reducing mental health symptoms, particularly anxiety (Kotera et al., 2022). The sailing adventure was organized by the tall ship professional crew. These two different, but long-time experiences in nature were undertaken by the participants during the academic fall semester, only weeks apart. The solo hikers had to prepare for cold temperatures including lots of rain, and even snow. The tall ship sailed in southern Europe, with mostly warm temperatures. However, temperature was low at the open sea. Based on presented literature, we propose that both the solo experience and the sailing adventure can enable growth-transcending outcomes by fostering psychological resilience in the direct engagement with nature. Although the context of the two experiences was very different, we assumed that they would share some common characteristics.

Research questions:

If psychological transformation occurred: How did informants increase their resilience during their long-time engagement in nature?

How were their experiences of resilience connected to transformative experiences within the green and blue environments?

Based on reviewed theory, we assumed three *a priori* themes that were explored deductively to understand underlaying structures of psychological transformation: 1. The need for reaching a mental low point of boredom or emptiness before growth (Torrissen and Løvoll 2025), 2. The need for time to reflect (Mau et al., 2021; Montserrat, 2020), and 3. The role of nature itself and of awe experiences (Naor and Mayseless, 2020a).

## Materials and methods

Two different qualitative data collection methods were used to explore psychological growth processes emerging from student practices. Qualitative interviews were performed a few months after the long-time engagement in nature, and written exams describing their experiences were analyzed. The material includes data from two very different contexts, and our ambition was to analyze the material to find commonalities across the contexts. We approached the material with a theory driven psychological perspective, in accordance with Template Analysis (Brooks et al., 2015), which is one way to perform Thematic Analysis. In accordance with the Template Analysis approach, the template was developed through coding and clustering themes in a hierarchical structure. This process was a back-and-forth process, ensuring that the data material was well covered in the Template. Using Template Analysis was suitable for analyzing similar as well as different qualities within the different contexts. In addition to using Template Analysis for the interview data, content analysis was performed on the informant’s written exams. Here, we were able to combine elements from the Template Analysis with logbook narratives and self-observations from the participants own systematic and daily wellbeing monitoring while on their trip.

### Participants

Two female (Eva and Anna) and two male students (Anders and Filip) participated in the research project. Participants were recruited from the ordinary BA program of students enrolled in the course “health promoting work” (20 ECTC), which is available for sports and outdoor education students during their third and final year of study. This course includes a mandatory practice, with students choosing between 1) making an 8-week health promotion program for a chosen target group with weekly meetings, 2) plan and carry out a long solo hike (maximum 14 days), or 3) volunteering in the social entrepreneurship program “Windjammer,” involving a 1-month sailing expedition. Selection to become a volunteer on the tall ship involved a recruitment interview performed by the Windjammer representatives. Practice is a core part of the BA program course, and students make their own research questions based on theory from the course, and they collect self-report and/or observational data throughout their practice. All students monitor self-selected indicators of wellbeing and write personal reflections relating to their own process and experience of participating in the practice. With 20–30 students attending the course each year, the distribution between those selecting the first and second options tends to be equal, while only a few choose the Windjammer option. For this research project, only students in the prolonged nature engagement practices (option 2 and 3) were invited to participate.

### Solo hikes in a forest-covered national park

Anders and Filip started their nature-exposure process by finding a relevant area for the solo hike. Detailed planning was essential in preparing for the hike, with particular attention to the risks associated with traveling alone. Landscape selection was also critical, and the University College imposed constraints related to steepness and overall risk exposure. Student plans were evaluated and approved by two independent teachers. They typically planned to hike 2–5 kilometers per day and to establish a campsite, either a timber shelter, originally built for forest workers and now available for hikers in the national park, or in tents. Following marked trails was preferred, but sometimes, these did not exist. They therefore needed to evaluate safety when crossing rivers, marshes, and steep terrain. During the hike, participants could engage in some mental exercises (described later), or they could simply remain open to what happened without any instruction. The outdoor study and nature guide students were trained in the international UIMLA standard prior to their solo hike. In other words, these students were trained to be outdoor leaders. Thus, they had extended experience with planning and undertaking hikes of 1–5-days´ duration. However, planning for 14 days without supplies was a new experience, and much of the planning revolved around food preparation. For safety reasons, each student carried an *Inreach*-device, making two-way satellite communication possible when out of mobile phone range. Participants also planned some “check in” communication with the teacher, to ensure safety during the hike.

While several landscapes were considered for the solo hike, Anders and Filip chose the same national park in Eastern Norway as their destination. This was mostly related to the fall climate, but other factors weighing in were the aesthetic qualities of the old pine forest, as well as access to primitive and cost-free shelters. This national park have few visitors during the autumn months, but one can typically meet reindeer herds. Encountering brown bears is possible, but very rare. This forest area is a natural habitat for foxes, hares, eagles, and infrequently also lynxes and wolverines. Some wolves might also pass by. Normally, these animals are very shy, and no special preparations were made other than keeping the *Inreach* technology communication systems operational. Students carried all their equipment with them to be self-sufficient, including tents and cooking gear. Use of two walking poles were compulsory when hiking to prevent sliding and falling.

Shinrin Yoku inspired invitations: For the solo experience participants, 14 separate invitations were made by the first author—one for each day. The invitations were kept in a waterproof bag, numbered, and enclosed with a staple. The purpose of the invitations was to inspire heightened awareness and self-reflection on the relationships with nature. Students were informed that these were invitations and not a compulsory part of the solo experience practice. However, to keep attention and wonder on sensory experiences, these invitations were meant to be supportive, especially during days with less motivational drive.

Here is one example:

Invitation day 2. Today you are invited to explore colors. Find a nice place where you can stand or sit. Allow your breathing to flow naturally right now. If you wish, you can lengthen your exhalation - exhale a little slower than you inhale. This will calm your body. Repeat three times. Now, focus on a field that is 1 × 1 meter in front of you. Maintain this focus for at least 10 minutes. Within this square, investigate how many different colors you see. How will you describe these colors? Choose your favorite color. You can play with your gaze - use both a focused "laser gaze" and soften your gaze. How does this affect the experience of colors? You are invited to write down your thoughts and feelings in your logbook.

### Windjammer: volunteering in a tall ship social entrepreneurship program

Eva and Anna signed up as volunteers for the 1-month social entrepreneurship program in the blue element. Preparations for the trip were given in digital meetings before the trip, where the students followed the procedures given by the tall ship management. Windjammer is a program offered to youths that need to reorient their lives. Often, they have dropped out of school or work, or they are at risk of dropping out. The youths were recruited from social services, and the Windjammer program is sponsored by both private and governmental organizations. The tall ship used in the project is a traditional fully rigged ship, which is operated manually. This means that participants, as part of the crew, need to climb in the rig, operate sails, as well as hundreds of ropes, perform sea watches, and steer the ship from a compass course, to mention a few things. Eva and Anna, in their roles as volunteers, supported the youths as they built competence and endurance, becoming familiar with sailing. However, this experience was also new to Eva and Anna, and their own growth process included learning practical seamanship and being part of the expedition, as well as being logged off internet and communication for a long period of time. All practical seamanship competence was organized by the professional team, and safety routines were strictly followed.

### Interviews

Interviews were conducted digitally through Zoom and recorded. The length of the interviews varied between 25 and 60 min. The mean length was 46 min. The recordings were saved digitally in “Nettskjema” and automatically transcribed through the AI function in this app. Subsequently, all transcriptions were reviewed and manually corrected, then safely stored with two-step authentication before deleting the voice files. The final transcribed material included 50 pages. All interviews were performed by the second author, which was the only time she was in contact with the students. The first author works with the BA education program of outdoor studies and nature guiding. She was not involved in recruitment or data collection. A semi-structured interview guide was used (see Appendix I).

### Written exam assignments

To maintain anonymity, the second author obtained the participants´ written exams. They were anonymized, then analyzed. The exam format was a home assignment resulting in a 4,000 word report of their chosen practice where students formulated and answered their own research question. The written exams were analyzed using content analysis to identify processes of growth.

### Interview analysis

Coding the material, the three *a priori* themes were very relevant. Several sub themes were developed in a further hierarchical structure, including four and five interpretation levels of the coding. Because the two practice situations (solo experience and Windjammer participation) were quite different, data analysis was performed in two phases. Although we believed that the three *a priori* themes were shared among both practice groups, we also wanted to include distinctions as well (see full template analysis in Appendix II). To identify different themes and where they came from, we separated themes from both groups (**bold**) from the themes from only the Windjammer group (normal) and those from only the solo experience (*italics*). The analysis was done in steps. First, the Windjammer interviews were analyzed. Secondly, the solo experiences interviews were analyzed. Thirdly, the whole interview material was re-analyzed across the two practices, solo trip and Windjammer, in search of common themes. This three-step process was performed to bring closeness to different contexts as well as understanding long-time engagement in nature as a more general process. The finished template included five overarching themes, 23 subthemes, with many of the subthemes being divided into one or two more sublevels.

### Ethics

The research project was approved by the national ethics committee, SIKT (application number 195444). The informant’s names are anonymized. The informants gave consent to participate in the research project involving both recording of interviews and giving permission to use their written exams for research purposes. Importantly, participation in the research project took place after the final grading, and the researchers had no influence on the grading process. The grades were not accessed for this research project.

## Results

Analysis of the material identified complex processes of psychological transformation across the different long-time engagements in nature. All four informants highly valued their long-time engagement in nature, and they all explained having obtained new perspectives adapting to difficult or challenging life experiences. This resulted in greater behavioral flexibility and the development of an inner dialog concerning life choices, values, and meaning. These responses were central to our subsequent analysis of resilience and transformative growth through complex experiences, first identified in student exam papers and later elaborated through interviews.

### Results from the self-monitoring of personal growth

Anders used a 12-item questionnaire of subjective wellbeing measures, including cognitive, affective, and eudaimonic measures, scored on a 0–10 Likert scale (Nes et al., 2018) for his 13-day solo experience. He collected data before the trip (T1), by the end of the trip (T2), and one week after the trip (T3). Here are his self-reported results (Table 1).

**Table 1:** Anders’s wellbeing monitoring.

Anders	T1 (before trip)	T2 (end of trip)	T3 (1 week after trip)
Cognitive dimension of satisfaction	7	7	8
Positive affect	6	9	6
Engagement	6	8	6
Negative affect	5	2	2
Meaning in life	8	7	9
How one contributes to other’s happiness and quality of life	7	7	8

In addition, general affective measures were reported every day, like “How do I feel today?”

**Figure 1:**
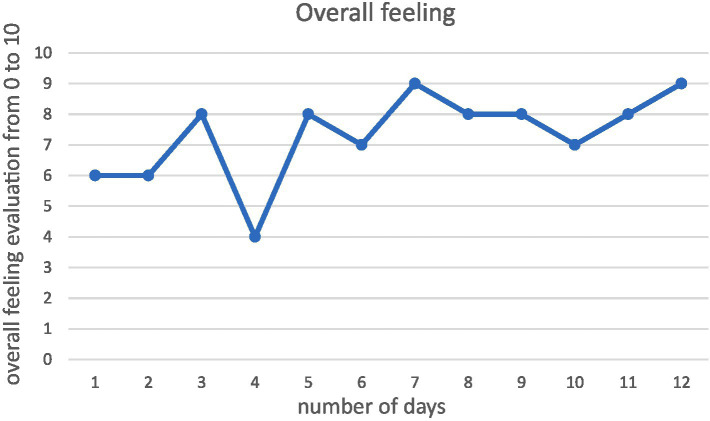
Anders’ overall positive/negative evaluation on daily basis.

On the daily report, there was a minimum score on day 4 (4), and the highest score (9) on day 7 as well as day 12 (Figure 1).

Anders also shared some quotes from his logbook. On day 4, he explained that the lack of goal and activity created restlessness and a “bad circle of negative thoughts mindsets.” He also explained what he called an “egodeath” in his own outdoor practice. This was explained as the challenge to trust his own practical skills without external support. On day 2, he experienced it being challenging to be alone, which indicated that this was a concern that started early during the trip. Nevertheless, Anders expressed happiness and satisfaction clearly 7 of 12 days in his logbook. This was particularly related to how he experienced nature as a place to spend his energy: “kilometers fly, and the mind brightens from movement.” To sum up, Anders reports: “I feel creativity that I have not been in contact with for several years. I feel I have set myself free again.” From his writings, he mentioned that he did not use the Shinrin Yoku invitations, and he also revised his plan to walk longer distances to feel comfortable doing solo hiking.

Filip also recorded self-reported wellbeing measures of his 13-day solo experience. Filip used 4 different time measurements: before trip (T1), mid-trip (T2), after trip (T3), and 1 week after trip (T4). See Table 2 for variables.

**Table 2:** Filip’s wellbeing monitoring.

Filip	T1 (before trip)	T2 (mid-trip)	T3 (after trip)	T4 (1 week after trip)
Cognitive dimension of satisfaction	6	7	6	8
Engagement	2	3	2	7
Happiness	8	4	5	7
Staying calm	1	8	6	5
Negative affect sum score	9	7	7	5
Meaning	5	7	5	8
Loneliness	6	8	7	2

Filip included the Shinrin Yoku invitations, which resulted in the development of poetic language in his logbook:

I think it was like each pine was a part of the same orchestra, and each needle was a musician in the orchestra. The wind was the conductor. The sporadic melodies were bird quarrels. They appeared suddenly, without foreboding and disappeared, just as fast.

Filip also reflected more, spending time in solitude:

If something, this trip so far has made me more unsure about life at home, future, career, socially. I get a lot of time to think, which surely is needed, that I find more new uncertainties and questions that I do not know the answers to…. yet.

He deepened with his feeling of loneliness:

To be alone on the trip… I find it challenging at times. […] I count down days, something I find too bad since I have been dreaming of and longing for this trip for very long. Maybe it didn’t turn out to be as romantic as I imagined.

Eva did not monitor her own personal growth process, but used an auto ethnographic approach to understand how the tall ship experience could support the growing processes of adolescents, by writing in her logbook. Using the framework “Five ways to wellbeing: Connect, be active, take notice, keep learning, and give” (Aked et al., 2008), she interpreted the work of practical seamanship and discussed how these different dimensions were part of the daily practices on the tall ship. She found that the five themes She found that the five themes were relevant to interpret much of what was already happening. Attention to these themes made more structured and conscious contributions to the work of the volunteers Eva learned a lot on the tall ship and got personal confirmation that work like this is something she wanted to continue later. She was very grateful for the experience, she made new friends, learned to master new skills, and enjoyed nature experiences like the starry heaven while on the tall ship.

Anna also analyzed the participant experiences through an autoethnographic method, and with the framework of “Five ways to wellbeing” to understand the supporting dynamics of adolescents. Anna decided to fully dedicate herself in the tall ship adventure. Every day, she wrote in her logbook. With her dedication to the work, she often was considered part of the professional crew among the participants. She found themes within “five ways of wellbeing” as structuring some important processes among adolescents, but she also used more of her own experience to understand the growth process. She mentioned that she learned a lot about practical skills, sailing, and the theory of sailing, but she also mentioned that she had been more self-aware and learned new life skills. She made new friends but also learned to pay attention to nature and bring attention to herself as well as others. She also explained that she learned from other participants when she herself was frightened. In the observation of the output from the participants, she mentioned that some had decided to actively engage in their social network and send a message to a friend upon the return home. Others wanted to complete high school, find a job, or actively find opportunities for the year to come. Some dared to be more authentic, some dared to be more assertive in social settings.

### Overarching themes from interviews

Through the analytic process, five overarching themes were identified: 1. Reaching a mental low point before growth, 2. Time to reflect, 3. Being present, 4. The role of nature and awe experiences and 5. Bodily rhythm and awareness. While the first four themes appeared in all interviews, the fifth theme was only articulated by the solo experience participants.

The sub theme of *reaching a mental low point before growth* was a solitude- and growth-related experience. Further common sub themes of solitude were freedom from mobile phones, turning inwards and finding your place in the new situation. As growth related experiences, the common sub theme was confirmation of life choices.

Common sub themes of *time to reflect* were new environment, new perspectives on life (and the subcategory becoming aware of strengths), and developing a growth mindset (and the subcategory of choosing a possibility mindset).

Common sub themes of *being present* were attention to special moments in nature, being fully attended to the tasks, and dedication to the project.

Common sub themes of *the role of nature and awe experiences* were peak nature moments, social awe experiences, and conversations about life. In both practices, meetings with wildlife made a strong impression. The category of own dreams appeared in both groups, where participants became more self-aware of the directions of study choices and work, often mentioned in relation to valuing such strong experiences.

The last overarching theme, *bodily rhythm and awareness* only appeared in the solo experience material. This theme was related to sub themes of sleep and hunger, change of original plan, and mood of discovery.

### From challenge to resilience informed by solo experience interviews

For both Anders and Filip, this was their first time alone for more than a few days. Both decided to finish after 13 days and then travel together back home. This journey involved carrying all necessary supplies for 2 weeks of camping in a heavy backpack, compounded by the cold and wet conditions of the autumn season. The overall experience was akin to a personal experiment, where individuals learned to thrive in their own company.

The dip, reaching a low point before growth, was clearly articulated among the solo hikers. It takes time to adjust to the new situation and to open up the mind to new experiences. For Anders, this low point was on day 4. In the interview, he thinks it takes 3–7 days to be able to shift the mindset. Then it is important to have more days to explore new ways of thinking and being. Filip mentioned his lower point on day 6 or 7. This was when the entertainment was finished (books and cards), and the snack was eaten. However, both informants found this challenging low point necessary for their growth. Moreover, it was not one single low point experience but several during the time alone. The feeling of being alone was difficult. Dealing with this difficulty and actively choosing to stay and to continue every day was an important aspect of the experience. In this way, their self-discipline was set into practice.

Adjusting cognitively to spending many days alone was challenging. It included the discovery of new routines, after 3–7 days of being alone, to change thought patterns. It was also followed by a worry about feeling lonesome, and the fact that it is hard to escape from one’s own thoughts. To deal with this, writing a diary and formulating thoughts and feelings was helpful. There were also glimpses of light during the days, bringing hope, but the informants were not prepared for how tough it turned out to be. One had to try hard to stay to the concept and accept feeling helpless without others and to miss feedback from others. In this situation, participants had to rely on themselves, considering the daily hiking plan, evaluating the weather conditions, their own energy level and capacity, and potential risks, to be able to make wise decisions. This was a feeling of unlimited freedom. Creativity emerged in different ways: Photographing, shouting and experimenting with sounds, making poetry, and craft making.

There were also feelings of emptiness, associated with restlessness, existential crisis, negative ongoing mindset, feeling finished even before the trip was over, which also challenged the willingness to stay. Finishing the adventure created a sense of vacuum. The solitude in nature led participants to seek new directions and by this, they also felt more responsibility, like feeling adults. As a personal reflection, they also mentioned the importance of staying true to themselves when choosing their educational direction, leading to meaningful work. Anders and Filip also experienced that their study choices and directions of work were confirmed. They became more aware of their personal needs for a good life, specifically recognizing the importance of social relationships and gaining clarity in what they truly desired in life. Dealing with restlessness involved acknowledging that there was always something to do, such as chopping wood, and just accepting restlessness as a natural bodily need, as expressed by Anders.

Solo hiking significantly impacted participants, contributing to personal growth through enhanced freedom and reflection. The solo hike facilitated a self-reflection process and a flexible mindset that would not have been achieved otherwise, leading to increased autonomy. Participants developed more positive attitudes when meeting new people and felt more connected to and more appreciative of nature. They also expressed increased gratitude and began to appreciate their close relationships at home in new ways. The experience provided new perspectives on life, serving as an important contrast to everyday life. Participants would recommend solo hiking to others, with a caveat about its potential toughness. However, despite many negative feelings during the hike, they would repeat the experience due to its positive outcomes, emphasizing the importance of the hike’s length and the opportunity to be alone for a prolonged period of time.

### From challenge to resilience informed by the tall ship adventure interviews

Eva and Anna commonly experienced seasickness, enduring its unpleasantness and the sensation of being thrown around in their berths. A prevalent challenge was the fear of climbing the rig, often stemming from a fear of heights, which typically took at least 1.5 weeks to familiarize oneself with. This fear was frequently overcome through distraction, such as sudden nature experiences like spotting dolphins, whales, or a star-filled sky. Additionally, mastering difficult tasks, such as packing and releasing sails from the highest position or operating at the most exposed part of the rig (“nåkken”), provided a means to conquer fear, often supported by mental encouragement from others. While motivations for learning varied among the participants, overcoming fear resulted in a pleasant feeling and a sense of being a valued crew member. Solitude was experienced during outlook watch and steering, intensified by the lack of mobile phone use, which sometimes led to sorrow over missing contact with close social networks.

Eva and Anna experienced a period of introspection, turning inwards to find their place within the new sailing environment, which involved a week without seeing land and the unique experience of “literally being in the same boat” with others. This new setting confirmed life choices and fostered a growth mindset, characterized by choosing a possibility-oriented attitude.

Being present was a significant finding, exemplified by feeling trust from both crew and fellow adolescents, and paying close attention to special moments in nature, such as shared sights of whales and dolphins. Participants demonstrated full dedication to their tasks and the overall project, with a strong desire to continue sailing even after the expedition concluded.

Nature and awe experiences played a crucial role, including peak nature moments like observing a star-filled sky, enjoying pleasant weather, and encountering dolphins and whales. Social awe experiences were also prominent, stemming from witnessing growth among youth, receiving support from windjammers in their own mastery, and engaging in meaningful conversations about life, including sharing personal dreams and those of fellow windjammers. These interactions fostered mutual understanding and led to the building of strong, lasting friendships characterized by a desire to stay together, and of shared moments of fun.

## Discussion

Both solo experiences and tall ship adventures, despite their differing contexts, share several common characteristics of psychological transformation, despite many negative moments of discomfort, anxiety, and feeling lonely. Participants described psychological resilience as emerging through sustained introspection and self-reflection in a sensory-rich environment, where the felt vastness of the open ocean or deep forest was a central element of the experience. Their experiences led to a deeper understanding of personal needs and desires. This transformation aligns with the flexibility hypotheses of healing (Hinton and Kirmayer, 2017) and with creative wellbeing (Torrissen and Løvoll 2025). All experiences were characterized by an emotional and existential low point, which participants found necessary for personal growth and a shift in mindset. Overcoming these difficulties by choosing a flexible mindset was a key aspect of both journeys. Furthermore, they emphasized the importance of being present in the moment, whether through dedicated focus on tasks or appreciating natural phenomena. A profound connection with nature and awe-inspiring experiences was also a shared theme, with instances like observing star-filled skies, pleasant weather, and encountering wildlife being highlighted as significant. Finally, both solo hikers and tall ship adventurers gained new perspectives on life, confirmed life choices, and expressed a desire to continue similar transformative activities, underscoring the positive long-term impact of their experiences.

The informants were faced with many different challenges during their adventures. Anders and Filip grappled with adjusting to prolonged solitude, dealing with loneliness, and confronting their own thoughts without external distractions. Their struggles involved a sense of helplessness without others and missing external feedback. They found coping activities in creative outlets like journaling, photography, and expressing themselves through voice or craft. The initial dip for solo participants was often characterized by restlessness and negative mindsets, underscoring the difficulty of adjusting to being truly alone with one’s thoughts. However, successful navigation of this solitude led to significant increases in autonomy, self-reliance, and a rekindling of creativity, aligning with research on the positive outcomes of chosen solitude (Naor and Mayseless, 2020a,b; Zelenski et al., 2013, 2021). The cognitive reappraisal of solitude, as explored by Rodriguez et al. (2020, 2023), likely played a role in transforming initial discomfort into a restorative experience for these participants. They adjusted the mindset to highlighting the importance of appreciating social relationships upon return. Nature could play a critical role in the development of solitude mindset over a loneliness mindset. In a recent population study of loneliness, nature connection was seen as having a moderating effect on social, emotional, and frequent experiences of loneliness (Hoff and Løvoll, 2026). It is likely that during the solo experience, connection to nature was strengthened, as seen in the poetry language from Filip. In this sense, nature becomes a partner, an active relationship, which is not reduced to only human relationships. Feeling invited to engage in relationships with nature, such as trees, plants, or atmospheres of the forest is then a two-way relationship, which then informs more about a solitude mindset than a loneliness mindset. Awareness to the specific context, by noticing daily change, tap into a mindset inspired by indigenous knowledge, nurturing a perspective of ecological sustainability and our dependence of nature (Spiegelaar, 2023).

Eva and Anna confronted physical discomforts such as seasickness and the fear of climbing the rig. Their challenges were often overcome through actively mastering difficult tasks and receiving mental support from a shared group. They felt less lonely as they inherently fostered immediate and continuous social bonding while being on adventure. Being “literally in the same boat” for an extended period led to supporting others’ development, forming friendships across different roles, and engaging in deep conversations about life and personal dreams. This collective experience of overcoming challenges together contributed to a strong sense of community and mutual growth. While moments of solitude and silence were powerful, such as moments alone steering the ship or doing lookout duty, watching in the bow of the ship, the primary growth trajectory was intertwined with collective effort and interpersonal dynamics. Participants confronted physical challenges like seasickness and fear of heights, but crucially, they overcame these with the direct mental and practical support of a shared group. This collective mastery fostered a strong sense of community, mutual understanding, and the development of friendships, resonating with findings on social awe experiences (Próchniak, 2022) and the therapeutic potential of shared engagement in nature (Brooke and Williams, 2021).

### The transformative power of immersion

Consistent with existing literature on nature’s role in personal growth (e.g., Mau et al., 2023a; Reiss, 2021), both solo hiking and tall ship volunteering served as potent catalysts for psychological resilience and personal transformation.

Furthermore, a pervasive theme across both groups was the importance of being present—fully engaged with tasks or attentively observing natural phenomena. This aligns with research highlighting the benefits of active noticing in nature (Richardson et al., 2022) and the integration of “being” and “doing” for holistic wellbeing (Pritchard et al., 2020). Moreover, the silence experienced, both during the solo experience and in silent moments on the tall ship was a source of inner dialog, what could also be described as an exploration of I-positions, articulated in the Dialogical Self Theory (Lehmann et al., 2019). The natural environments provided a unique backdrop for this heightened presence, fostering a deeper connection to nature and a sense of awe (MacKerron and Mourato, 2013; Hakoköngäs and Puhakka, 2023). For Filip, the Shinrin Yoku invitations moved forward this process, while for Anders, he felt enough stimulated without these invitations. Anders moreover felt a need for more activity than initially planned for, while Filip was satisfied with hiking smaller distances. This resonates with Próchniak (2022) and the view that there are different personality profiles in baseline satisfaction with life, control of life, meaning of life, and positive emotions, pointing to the preference of different outdoor stimuli, such as hard versus soft adventure hikes.

Both long-time engagements in nature contributed to participants’ eudaimonic wellbeing—a sense of purpose, meaning, and personal fulfillment (Ritpanitchajchaval et al., 2023). This meaning emerged from confronting existential questions and affirming life choices in the absence of external “facades” (Reiss, 2021). It also stemmed often from supporting the growth of others, mastering complex tasks collectively, and contributing to a shared endeavor, as seen by Anna and Eva. The experiences fostered a heightened sense of autonomy, whether through self-reliance in the wilderness or active participation and trust within a collaborative crew (Sheldon et al., 2021).

Ultimately, these findings underscore the diverse pathways to personal growth offered by extended engagement in nature. They leverage the inherent qualities of natural environments—their ability to inspire awe, demand presence, and provide a context for navigating discomfort—as fundamental ingredients for fostering psychological resilience and transformation. While our findings align with those of a Canadian outdoor study—conducted through regular daytime place-reconnection practices—which reported reduced stress, anxiety, and feelings of isolation, alongside increased calmness, joy, mindfulness, cognitive clarity, and a strengthened sense of healing and emotional resilience (Spiegelaar, 2023), the intense inner journey of solo hike and tall ship sailing add psychological resilience to be a powerful part of the psychological transformation. Also, in bringing in the vastness of the ocean and remote forest, the existential question of “who am I in this world” becomes an urgent question.

### Limitations and future research

This study is limited by the number of participants. More informants could add more variations and nuances to the main narrative. The findings across context moreover point to some intersubjective associations with long-time engagement in nature. Even though multiple qualitative methods were used, such as self-reports, logbooks student exams and interviews, additional methods could shed light over the context, such as videos, landscape analysis, using geographic information system (GIS) reports, weather reports, or other sources of deepening contextual information, as well as testing for personality factors and other baseline measures. The use of Template analysis seemed to be fruitful, where *a priori* themes were identified, as well as new themes were developed in the material. Other qualitative analysis could demonstrate more or different qualities. There might be differences between cultures and natural environments in the understanding of safety and relevance for long-time engagement in nature. For example, in Norway, it is regarded as safe to sleep in the forest and to hike alone in non-alpine areas if well prepared for it.

The qualitative investigation of psychological resilience during long-time engagement in nature adds important insights beyond quantitative research. The context sensitive bottom-up perspective gives voice to painful and vulnerable situations, and how a flexible mindset can make a change, adapting to the circumstances. Nature plays a vital role within these processes, as being a relational partner, offering silence, imagination, and hope. More research is needed within this scope, across different ways of engaging in nature, as well as within different cultures. Direct monitoring of opportunity feelings, such as interest, awe, wonder, and love (Torrissen and Løvoll, 2025) could display even richer approaches to understand the depth of the experience. Recently, associations were found between character strengths and nature connectedness (Passmore et al., 2025). Future studies of long-time engagement in nature would benefit from including character strengths in the perception of loneliness versus solitude in nature.

Relating to gender, two males chose the solitude in nature, and two females chose the Windjammer adventure. We do not have enough data to say if the chosen practice is flavored by gender. Other years, female students also chose the solo adventure, and males chose to be voluntary workers in the Windjammer program. There were no gender separation in sleeping arrangements (hammocks and berths) also how sleeping was organized in hammocks and berths. Interestingly, there were also female officers and role models among the professional crew. These gender- and safety conditions might be very different across the world and would certainly influence the results. There is a need for more full-scale studies but also cultural comparative studies to understand the potential of solo hikes and tall ship adventures in long-time engagement in nature to identify safe and generalizable practices for these.

## Conclusion

This study demonstrates that both solo nature immersion and socially engaged nature adventures serve as powerful catalysts for increased psychological resilience and transcending growth. Despite many negative feelings, the adventures foster autonomy, self-reliance, and deep introspection by challenging individuals to confront themselves in solitude, as well as shared mastery, mutual support, and profound social bonding as sources of growth. Across both contexts, a fundamental process involves navigating a “dip” or challenging low point, which ultimately leads to a shift in mindset and enhanced resilience. The consistent emphasis on presence and the experience of awe in nature underscore the environment’s inherent capacity to facilitate these transformative journeys. Ultimately, these findings highlight that prolonged engagement with nature, whether alone or in a group, offers diverse, yet potent, pathways for individuals to gain new life perspectives, confirm choices, and cultivate a deeper sense of purpose and wellbeing. How a situation of being alone in nature is perceived is essential, giving rise to a solitude mindset over a loneliness mindset, which is something that can be cultivated and trained. Being in nature, here conveyed by intense moments of vastness and immersion, provides a possibility of developing a stronger connection to nature. This is essential in providing a mindshift toward more ecological ways of living.

## Data Availability

The raw data supporting the conclusions of this article will be available upon request.
