# Supplementary material for: Silence to grow: psychological transformation during long-time engagement in green and blue nature
Source: Front Psychol. 2026 Mar 16;17:1652406. doi: 10.3389/fpsyg.2026.1652406 (PMC13034435; doi:10.3389/fpsyg.2026.1652406)
Supplement: Supplementary file 1 [file Table_1.DOCX]

**Appendix I**

The interviews used semi-structural interview guide, with the following questions:

1.What kind of trip did you choose? What was your background or intention for choosing this particular trip / trail?

2. What expectations did you have for the trip before you went? (about being alone in nature / what you expected to be personal challenges).

3. What were the most important highlights for you on the trip? These can be both positive and negative.

4. What experiences or highlights contributed to you changing your perspectives? (on life / on nature / on yourself).

5. What have you learned about yourself through this trip?

6. There is a lot of talk about BEING in nature rather than DOING in nature. What does this mean to you? Did you have any experience with this on the trip?

7. How did you experience collecting data about your own experiences as systematically as you did on this trip? Has this made you more aware or contributed to changes in: a. Your relationship with nature b. Your relationship with yourself c. Your own acceptance of yourself d. Meaning of life

8. What "peak experiences" did you have on the trip?

9. What was the most demanding thing you experienced? How has that experience affected you (your view of yourself / what you can achieve / master)

10. Anything else you would like to tell us?
