# Supplementary material for: Silence to grow: psychological transformation during long-time engagement in green and blue nature
Source: Front Psychol. 2026 Mar 16;17:1652406. doi: 10.3389/fpsyg.2026.1652406 (PMC13034435; doi:10.3389/fpsyg.2026.1652406)
Supplement: Supplementary file 2 [file Table_2.DOCX]

**Appendix II: Template**

Normal: Windjammer participants, *Italics: Solo hikes*, **Bold: Both groups**

1. **To reach a mental bottom line of boredom or emptiness before growth**
   1. Seasickness
      1. Common experience
      2. Endurance
      3. Unpleasantness
      4. To be thrown around in the berth
   2. Fear of climbing the rig

1.2.1 Fear of heights

1.2.2 To familiarize climbing the rig takes at least 1 ½ week

1.2.3 To overcome fear through other attention

1.2.3.1 Sudden nature experience

1.2.3.1.1 Dolphines

1.2.3.1.1 Whales

1.2.3.1.1 Star heaven

1.2.3.1 Solve a difficult task

1.2.3.1.1 Pack and release sails in the highest position

1.2.3.1.2 Operating at the most exposed place in the end of

the rig (“nåkken”)

1.2.3.1.3 Mental support from others

1.2.4 Different motivation to learn among windjammers

1.2.5 Pleasant feeling to overcome the fear and become a valued crew member

**1.3. Solitude**

1.3.1 Alone on the sea guard watch and when steering

**1.3.2 No use of mobile phones**

1.3.2.1 Sorrow to miss contact with close social network

*1.3.2.2 Fear of home-length if using it*

**1.3.3 To turn inwards**

**1.3.4 To find your place in the new situation**

1.3.4.1 Adjust to the rhythm of the tall ship

*1.3.4.2 Adjust to days being alone*

*1.3.4.2.1 Discover new routines*

*1.3.4.2.2 The need of staying more than 3-7 days solo hiking*

*to change thought patterns*

*1.3.5 The fright of feeling lonesome*

*1.3.5.1 Worry to feel bored*

*1.3.5.2 Hard to escape from own thoughts*

*1.3.5.3 Writing diary to formulate thoughts and feelings*

*1.3.5.4 Small glimpses of light*

*1.3.5.5 Not prepared to how tough it turned out to be*

*1.3.5.6 Tried hard to stay to the concept*

*1.3.5.7 Missed feedback for my thoughts from others*

*1.3.5.8 Feeling helplessness without others*

*1.3.6 The feeling of being one’s own chief*

*1.3.6.1 The feeling to be able to choose direction*

*1.3.7 Unlimitedness freedom*

*1.3.8 Creativity*

*1.3.8.1 Photographing*

*1.3.8.2 Using voice: Shout out and feel released*

*1.3.8.3 Making poetry*

*1.3.8.4 Craft making*

*1.4. Emptiness*

*1.4.1 Restless. Too much energy*

*1.4.2 Existential crisis*

*1.4.3 Negative ongoing mindset*

*1.4.4 To feel finished, before the end of the trip*

*1.4.5 Choosing to stay despite empty feelings*

*1.5 Downward spiral of surplus*

*1.5.1 When finished the establishment of making new routines*

*1.5.2 When finished entertainment, like reading and playing cards*

*1.5.3 When finished snack*

*1.5.4 Reaching bottom line day 6-7*

*1.5.5 There were many bottom-line experiences throughout the solo hike*

**1.6 Growth related experiences**

*1.6.1 Feel more like an adult*

*1.6.1.1 Entering the vacuum of finishing studies and see a new*

*direction*

**1.6.2 Confirming life choices**

*1.6.2.1 Right to follow the intuition of starting a new education*

*1.6.2.2 Seek for work with meaning*

*1.6.2.2.1 It is meaningful to live a life as good as possible*

*1.6.2.2.2 Must remember to prioritize from this insight*

1.6.2.2 Enjoy working with people in nature setting

*1.6.3 Becoming aware personal needs for a good life*

*1.6.3.1 Awareness of the need of social relationships*

*1.6.3.2 Awareness of what I want in life*

*1.6.4 Dealing with restlessness*

*1.6.4.1 There is always something to do*

*1.6.4.1.1 Chopping wood*

*1.6.4.2 Accepting restlessness as body need*

*1.6.5 The effect of solo hiking*

*1.6.5.1 Freedom and reflection contributed to personal growth*

*1.6.5.1.1 The hike released the self-reflection process*

*1.6.5.1.2 Would not achieve the same benefits without this solo*

*1.6.5.2 More autonomy*

*1.6.5.3 More positive attitude meeting new people*

*1.6.5.4 More connected and appreciative to nature*

*1.6.5.5 More grateful*

*1.6.5.6 Appreciate the people at home in new ways*

*1.6.5.7 Got new perspectives on life*

*1.6.5.8 Important contrast to everyday life*

*1.6.5.9 I would recommend this to others, but be prepared how tough*

*it might be*

*1.6.5.10 I would do it again in the same way. It was a positive result.*

*1.6.5.10.1 The length is important*

*1.6.5.10.2 Needs to be away from many people*

1. **Time to reflect**

**2.1 New environment**

2.1.1 One week without seeing land

2.1.2 Literally being in the same boat

2.1.3 Supporting others in their development process

2.1.3.1 Adolescents in the social work setting

2.1.3.2 Development of friendships across roles

*2.1.4* *Until fourteen days to fill in a new forest national park area*

*2.1.5 Learning to be in one’s own company*

*2.1.6 First time solo experience for more than a couple of days*

*2.1.7 Carrying all needs for 14 days of camping in a heavy backpack*

*2.1.7.1 Autumn season, being cold and wet climate*

*2.1.8 Like being in a personal experiment*

**2.2 New perspectives on life**

**2.2.1 Becoming aware of strengths**

2.2.2 Becoming aware new possibilities through role models

**2.3 Developing a growth mindset**

**2.4.1 Attitude: choose a possibility mindset**

1. **Being present**

3.1 Choosing to drop exam to be more present

3.2 Feeling trust from the crew as well as the windjammers

**3.3** **Attention to special moments in nature**

3.3.1 Shared moments seeing whales and dolpins

*3.3.2 Changes and surprises in the landscape*

**3.4 Being fully attended to the tasks**

*3.4.1 Able to sit and be concentrated for half an hour*

*3.4.2 Use of tobacco pipe to stay calmed*

*3.4.3 Mindful self-care*

*3.4.3.1 Tooth brushing*

*3.4.3.2 Washing the body*

3.4.4 Being mindful to use skills to solve the sailing tasks

3.5 Enjoying moments of sailing

**3.6 A dedication to the project**

3.6.1 Wish to continue the sailing, also after the end of expedition

*3.6.2 Wish to stay the whole period of 14 days for the solo experience*

*3.7 Being oriented to just “being along the way”*

*3.8 Using tools: Shinrin Yoku inspired invitations*

*3.8.1 Dropped these invitations immediately*

*3.8.1.1 Wished to move away from obligations and structures*

*3.8.2 Found the invitations to be very powerful*

*3.8.2.1 Some invitations were weird*

*3.8.2.2 Liked best the “listening exercise”*

*3.8.2.3 A way of forcing attention to be focused*

*3.8.2.4 Was moved out of my comfort zone*

*3.8.2.4 Were “sucked” into the invitations*

*3.8.2.5 Got more aware about tiny nuances in nature*

*3.8.2.6 A meaningful way of filling the time*

*3.9 Moving perspective from a distanced perspective to being a part of nature*

*3.9.1 Ontological experience of “being warmed up” by nature*

*3.9.2 Awareness of ambivalences in approaches to nature*

*3.9.2.1 Being in a guide role*

*3.9.2.2 Personal needs and satisfaction*

*3.9.3 Feeling more aware and connected to details in nature*

*3.9.4 Awareness of different ways of being in nature influence our relationship to nature*

1. **The role of nature and awe experiences**

**4.1 Peak nature moments**

4.1.1 Star heaven

4.1.2 Nice weather

4.1.3 Dolphines and whales

*4.1.4 Surprising experiences*

*4.1.4.1 Reindeer herds approached with sounds and vibration*

*4.1.4.2 Huskies turned up while chopping wood*

*4.1.4.3 New scenery*

*4.1.4.4 The surprise of finding new shelters*

*4.1.5 Pleasant experiences*

*4.1.5.1 Feeling closeness to the reindeer herds*

*4.1.5.2.1 The feeling of calmness*

**4.2** **Social awe experiences**

4.2.1 Seeing growth among youths

4.2.2 Support from windjammers in own mastering

**4.2.3 Conversations about life**

4.2.3.1 Dreams of Windjammers

**4.2.3.2 Own dreams**

*4.2.3.2.1. Self-talk about life*

4.2.4 To get each other to know

4.2.5 Building friendships

4.2.5.1 Staying together

4.2.5.2 Fun

*4.2.6 Appreciate friends and social network*

*4.2.7 Becoming more aware social relationships*

1. *Bodily rhythm and awareness*

*5.1 To be aware the bodily needs of sleep and hunger*

*5.1.1 Not using alarm clock*

*5.1.2 Pleasant to find routines based on the body needs*

*5.2 Change the original hiking plan*

*5.2.1 More satisfactory to hike longer distances*

*5.2.2 Finding new shelters based on weather and people’s suggestions*

*5.3 Mood of discovery*

*5.3.1 To find the childish self*

*5.3.2 To follow the curiosity of wonder*

*5.3.2.1 Hike outside the path*

*5.3.2.2 Follow intuition when seeing signs*

*5.3.2.2.1 Finding fox holes*
